# Characterization of Cotton Ball-like Au/ZnO Photocatalyst Synthesized in a Micro-Reactor

**DOI:** 10.3390/mi9070322

**Published:** 2018-06-26

**Authors:** Ki-Joong Kim, Peter B. Kreider, Ho-Geun Ahn, Chih-Hung Chang

**Affiliations:** 1 National Energy Technology Laboratory (NETL), U. S. Department of Energy, 626 Cochrans Mill Road, Pittsburgh, PA 15236, USA; 2 School of Chemical, Biological, and Environmental Engineering, Oregon State University, Corvallis, OR 97331, USA; 3 Research School of Engineering, The Australian National University, Canberra, ACT 2602, Australia; peter.kreider@anu.edu.au; 4 Department of Chemical Engineering, Sunchon National University, 255 Jungang-ro, Suncheon 57922, Korea; hgahn@sunchon.ac.kr

**Keywords:** continuous photocatalyst synthesis, core-shell structure characterization, Au/ZnO photocatalysts, cotton ball-like Au/ZnO

## Abstract

Noble metal/metal oxide nanostructures are an efficient system in photocatalysis. Continuous and scalable production of advanced particle systems will be a requirement for commercial-scale deployment for many applications, including photocatalysis. In this work, Au/ZnO structures were synthesized in a continuous flow micro-reactor at room temperature and the detailed characteristics of the product indicate a specific cotton ball-like core-shell microstructure that showcases specific advantages compared to traditional batch synthesis methods. The formation pathway of the core-shell Au/ZnO structures is discussed with the pH-dependent speciation diagram, and photocatalytic activity was assessed under simulated sunlight, demonstrating the enhanced performance of the cotton ball-like Au/ZnO microstructures in photocatalytic dye degradation. This work describes the application of microreaction technology in the continuous production of metal/metal oxide photocatalysts.

## 1. Introduction

Incorporating noble metal nanoparticles (NPs) into semiconducting metal oxides, such as TiO_2_ and ZnO, is a good approach for enhancing the catalytic activity in photocatalysts because it promotes interfacial charge-transfer processes [1,2,3,4,5,6,7,8]. In particular, many efforts have studied the controlled synthesis of core/shell structured composites, which can significantly enhance the photocatalytic activity by decreasing the recombination rate of the photogenerated electron-hole pairs (or excitons) within the semiconductor particles [9,10,11]. These NPs are generally synthesized in small batch reactors with low production rates. The demand for large-scale production of these advanced structures is increasing due to several factors, including for use in commercial applications. However, large-scale syntheses have difficulty in precisely controlling reaction conditions. Especially, continuous synthesis is very challenging for core/shell structured photocatalysts, without losing their unique properties [12,13,14].

Nanomaterial synthesis in a micro-reactor with precisely controlled reaction conditions (i.e., minimize heat/mass transfer by instantaneous mixing) has become a versatile approach in modern synthetic chemistry [15,16] and been developed for producing metals (Au, Ag) [17,18,19,20,21], metal oxides (ZnO, TiO_2_, Fe_3_O_4_, SiO_2_) [22,23,24,25], chalcogenides (CdSe, PbSe, CuInSe_2_) [26,27,28], and core-shell (Fe_2_O_3_/SiO_2_, CdSe/ZnS) NPs [29,30]. These methods should be amenable to developing an industrially-relevant large-scale synthesis method by arraying many micro-reactors in parallel (hundreds to millions) [31]. Furthermore, micro-reactor synthesis is considered a simple, convenient, low cost, and environmentally friendly synthetic method under mild conditions [32].

Au NPs embedded in ZnO (Au/ZnO), which is regarded as a representative photocatalytic material due to its efficient absorption of light in the visible region, was used as a model system in this work to demonstrate the viability of micro-reactor synthesis for technological applications. There are many works that have reported core-shell [33,34,35] or hierarchical [36,37,38,39,40] Au/ZnO structures; none of these attempted a continuous process for scalable synthesis.

To the best our knowledge, this is the first report that produces a cotton ball-like, core-shell Au/ZnO photocatalyst using a micro-reactor under hydrothermal conditions, which would provide a convenient means to increase production rates to commercial scales. Detailed characteristics of the product are discussed with various techniques, thereby providing a possible mechanism regarding the formation of a core-shell Au/ZnO structure in a micro-reactor. Furthermore, the photocatalytic activities of Au/ZnO were investigated for dye degradation under visible-light irradiation.

## 2. Materials and Methods

### 2.1. Synthetic Procedure

Au/ZnO photocatalyst was synthesized in a micro-reactor, which consisted of feeding, nucleation, and heating zones [41]. The precursors, a mixture solution of Zn(NO_3_)_2_·6H_2_O (0.05 M, Sigma-Aldrich, St. Louis, MO, USA) and HAuCl_4_·6H_2_O (4.2 × 10^−4^ M, STREM Chem., Newburyport, MA, USA), were initially pumped into the Tygon tubing (1.59 mm i.d., Upchurch Scientific, Silsden, UK) individually at a flow rate of 6.5 mL·min^−1^ via a peristaltic pump (REGLO Digital, Ismatec, Wertheim, Germany), then mixed with Na_2_CO_3_ (0.1 M) as an agent for pH control in a micro T-mixer (0.50 mm i.d., Upchurch Scientific, Silsden, UK). The resulting mixture, with a 6620 cm·min^−1^ of linear velocity, was passed through a growing zone of 1 m long coil and kept immersed in an oil bath maintained at 80 °C. The resulting precipitate was collected in a beaker. After 1 h, the supernatant was decanted, filtered (No. 1, Whatman, Maidstone, UK), and washed with warm deionized water 5 times. The product was dried at room temperature for 24 h and aged at 100 °C for 12 h, followed by calcination in air at 500 °C for 5 h. For comparison, Au-ZnO with similar Au contents was also prepared using the co-precipitation method (Au-ZnO-CP), according to our previous work [42]. Briefly, an aqueous solution of 1 M Na_2_CO_3_ solution was slowly dropped into the mixture solution of Zn(NO_3_)_2_·6H_2_O and HAuCl_4_·6H_2_O with continuous stirring at 80 °C. The resulting precipitate was filtered, washed, dried, and calcined.

### 2.2. Characterization

Scanning electron microscope (SEM) analysis was conducted by Quanta 600 (FEI, Hillsboro, OR, USA) using 15–20 kV accelerating voltage in conjunction with energy dispersive spectroscopy (EDS). The crystalline phases were identified by D8 Discover X-ray diffraction (XRD, Bruker, Billerica, MA, USA) operating at 40 keV and a current of 40 mA with Cu kα radiation (0.154 nm) in the 2θ scan range from 30° to 70° with a step size of 0.05°. X-ray photoelectron spectroscopy (XPS) was performed on a ESCALAB 250 (Thermo Scientific, Grand Island, NY, USA) with a monochromatized Al Kα X-ray source (1486.6 eV) and with a 500 μm spot size. The binding energy was calibrated using the C 1s signal located at 284.8 eV. Time-of-flight secondary ion mass spectrometry (ToF-SIMS) spectrum was collected using an ION TOF IV instrument (ION TOF, Heisenbergstraße, Münster, Germany) with 25 keV pulsed Bi^3+^ primary ion gun at a 10 kHz repetition rate and an average in the current of 0.5 pA. High resolution transmission electron microscopy (HRTEM) and high-angle annular dark-field scanning TEM (HAADF-STEM) were performed by a FEI Titan FEG-TEM (FEI, Hillsboro, OR, USA) at an accelerating voltage of 300 keV. The loading content of Au in the Au/ZnO was observed by using a D-TIME 3000 DC inductively coupled plasma-atom emission spectrometry (ICP-AES, Perkin Elmer, Waltham, MA, USA). The BET specific surface areas were measured at −196 °C using an ASAP 2010 analyzer (Micromeritics, Norcross, GA, USA). The ultraviolet-visible (UV-Vis) absorption spectrum was obtained by using a V-670 UV-Vis-NIR spectrophotometer (Jasco, Easton, MD, USA).

### 2.3. Photocatalytic Test

Photocatalytic activities of samples were evaluated by the degradation of methylene blue (MB) solution under simulated sunlight. A 500 W Xe lamp (UXL-16B, Ushio, Tokyo, Japan) was used as a light source, with a maximum intensity of 494 nm. The spectrum of incident light was obtained by USB 2000 fiber optic spectrometer (Ocean Optics, Dunedin, FL, USA). The reactor was set 20 cm away from the output beam. In a typical experiment, 0.1 g of the sample was added into 100 mL of 10 mg·L^−1^ MB solution.

Prior to photocatalytic testing, the solution was stirred for 30 min in darkness to reach the adsorption-desorption equilibrium of MB to avoid any error from the initial adsorption effect, then irradiated with stirring. At different irradiation time intervals, solutions were taken from the reaction suspension, filtered through a 0.45 µm Millipore filter to remove the particles, and then analyzed for MB content using a UV-Vis-NIR spectrophotometer (V-670, Jasco, Oklahoma City, OK, USA) at 664 nm. The rate of degradation was calculated by assuming pseudo-first-order kinetics and, hence, the rate constant for the degradation, k, was obtained from the first-order plot, according to the following equation: ln(C/C_0_) = kt, where C_0_ is the initial absorbance of MB, C is the absorbance of MB after a time (t), and k is the first-order rate constant.

## 3. Results and Discussion

### 3.1. Characteristics of Au/ZnO

**Figure 1:**
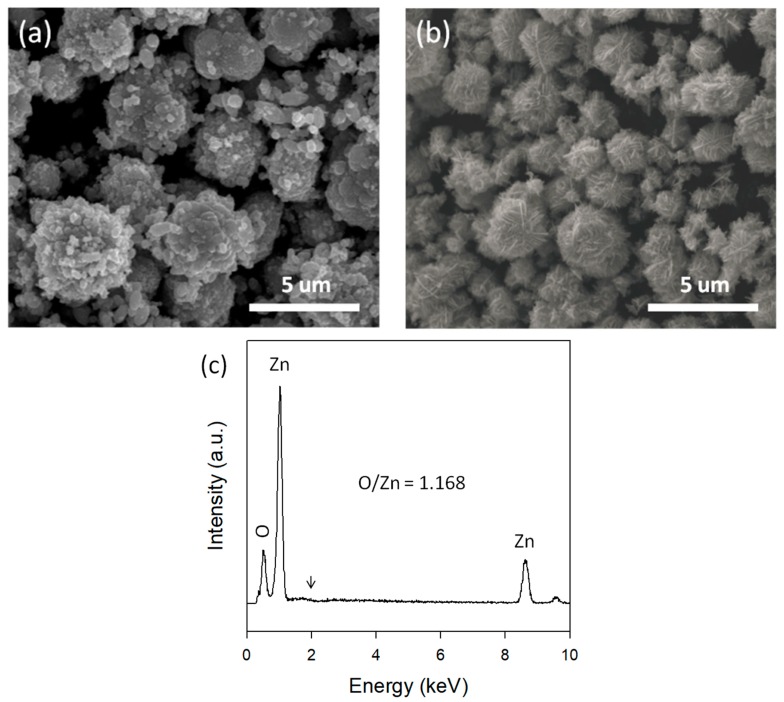
SEM images of (**a**) Au-ZnO-CP and (**b**) Au/ZnO synthesized in a micro-reactor. (**c**) EDS spectrum of Au/ZnO synthesized in a micro-reactor (arrow at ~2.2 keV represents the X-ray signals of the Au-L1).

The morphology of samples was observed by SEM. As shown in Figure 1a, Au-ZnO-CP shows popcorn ball-like Au-ZnO particles. In contrast, the Au/ZnO synthesized in a micro-reactor reveals a cotton ball-like structure with a diameter of 1–4 micrometers (Figure 1b), which is assembled by a large number of interconnected nanosheets. From the EDS spectrum (Figure 1c), only Zn and O were detected, while no Au was detected within the detection limits over the Au/ZnO sample synthesized in a micro-reactor. The atomic ratio of O/Zn is 1.168, revealing the Zn deficiency in the Au/ZnO surface structure. The Zn deficiencies are presumably attributed to the hydroxyl groups and adsorbed water on the ZnO surface.

**Figure 2:**
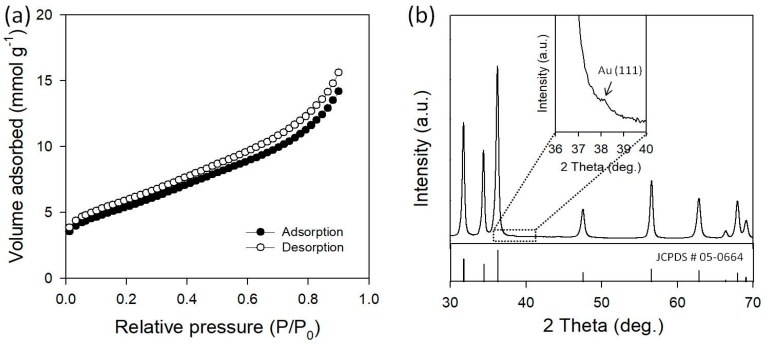
(**a**) Adsorption-desorption isotherm and (**b**) XRD pattern of Au/ZnO synthesized in a micro-reactor (Inset of magnified XRD pattern for Au (111) and JCPDS No. 05-0664 of hexagonal ZnO crystal).

Texture properties were characterized by the nitrogen adsorption-desorption process at −196 °C, as shown in Figure 2a. The Au/ZnO presents a type II isotherm, which is characteristic of macroporous structures. Au/ZnO presents a larger specific surface area of 21.7 m^2^·g^−1^, compared with that of the flower-like pure ZnO (16.6 m^2^·g^−1^) synthesized in a micro-reactor in similar synthesis conditions [41]. Typically, low dimensional nanostructures, such as nanoparticles, nanorods, nanosheets, etc., were found to have a small surface area of <10 m^2^·g^−1^ [43], indicating that the cotton ball-like Au/ZnO particles would provide more active surface area as photocatalysts. Figure 2b shows the XRD pattern of Au/ZnO. The diffraction peaks for Au/ZnO can mainly be indexed as the hexagonal phase ZnO (JCPDS No. 05-0664). The XRD results showed that the formation of a new alloy form was not observed in Au/ZnO. The strongest Au peak should be located at 38.2° for the Au (111) plane, but there is only an extremely small intensity peak at this location (inset of Figure 2b). This may be due to extremely small Au NPs in the ZnO macrostructure or because the peak is obscured by the tail of the ZnO (101) peak at 36.5°. From the Scherrer equation, the crystallite size of Au NPs was estimated to be ~3.4 nm. It is a possibility that Au NPs are surrounded by ZnO with a thickness of more than 1 um, which may exceed the penetration depth of X-rays, resulting in the missing signals of Au in the XRD patterns.

**Figure 3:**
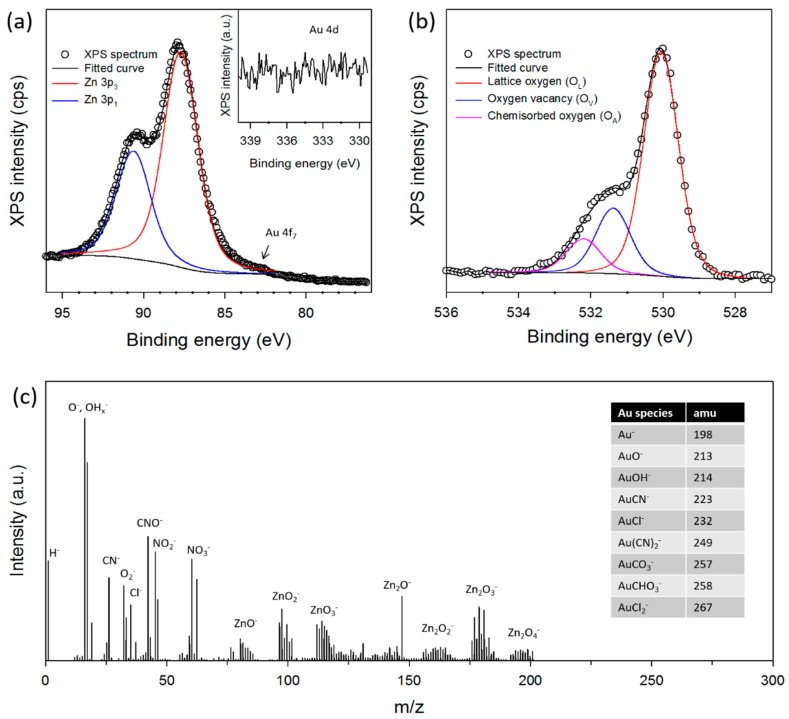
XPS spectra of (**a**) Zn 3p (insert of Au 4d) and (**b**) O 1s, and (**c**) negative ToF-SIMS spectrum of cotton ball-like Au/ZnO synthesized in a micro-reactor. Insert in Figure 3c shows negative Au species for reference.

Therefore, material composition, as a function of depth, is a key factor in getting the elemental information needed to help us to understand the structure of Au/ZnO synthesized in a micro-reactor. The ideal tool for analyzing surface composition is XPS, which is only sensitive to the first 10 nm or less. The Au 4f, Zn 3p, and O 1s of XPS spectra are presented in Figure 3. The most intense peak of Au arises from the Au 4f spectrum, but this energy region partially overlaps with that of the Zn 3p spectrum [44], as shown in Figure 3a. For this reason, we considered the Au 4d peaks (insert in Figure 3a), which are not as strong as the Au 4f peaks, but are stronger than the other Au peaks. The strongest Au 4d peaks must appear at around 334 eV, but were not detected in this region.

In addition, the chemical state of O is very important for photocatalytic materials. The XPS spectrum of O 1s for Au/ZnO is shown in Figure 3b. The peak deconvolution of the O 1s lines also gives more information about the relative amount of different surface oxygen species. The O 1s peak can be fitted into three Gaussian peaks located at 532.2 eV, 531.4 eV, and 530.1 eV, respectively, indicating three different kinds of O species in Au/ZnO. The higher O peak at 530.1 eV can be ascribed to the lattice O (O_L_) in ZnO and the medium O peak at 531.4 eV is associated with O^2−^ ions in O vacancy (O_V_) regions within the matrix of ZnO. The lower O peak at 532.2 eV is usually attributed to chemisorbed oxygen (O_A_) species, such as CO_3_, adsorbed H_2_O, or adsorbed O_2_ [45,46,47,48]. The atomic ratio of total O (O_T_) to Zn on the surface of Au/ZnO is 1.163, which is in very good agreement with the EDS analysis (Figure 2b). On the other hand, the calculated O_L_/Zn ratio is 0.805, which reveals an O vacancy on the crystal lattice on the surface of the sample. This O vacancy could promote the visible-light absorption of the sample, leading to high photocatalytic activities towards the degradation of organic dyes.

To investigate the chemical composition of Au/ZnO in greater detail, we performed a ToF-SIMS analysis, which is about two orders of magnitude more sensitive than XPS and can, potentially, confirm a very small amount of Au species on the ZnO surface. Figure 3c shows the negative ToF-SIMS spectrum up to *m*/*z* = 300 amu. The mass spectra reveal no clear signals for Au clusters that should appear at above 200 amu (see the table in Figure 3c). This indicates that there is no presence of Au ions on the surface of ZnO. It is well known that the ToF-SIMS method is used to characterize the surface of the sample and the detectable penetration depth is less than 5 nm. Therefore, the presence of Au may not also be detected by XPS and ToF-SIMS when the Au is embedded in a thick ZnO shell. Nevertheless, the total amount of Au ions in Au/ZnO was confirmed by an ICP-AES and 2.4 wt % was obtained. This suggests that Au NPs are embedded into the ZnO, core-shell structure Au/ZnO. It is believed that this core-shell Au/ZnO could play a key role in the photocatalytic activity due to the presence of abundant metal-semiconductor interface that can effectively transfer the photo-excited electron from the valence band to the conduction band in ZnO more efficiently [49].

**Figure 4:**
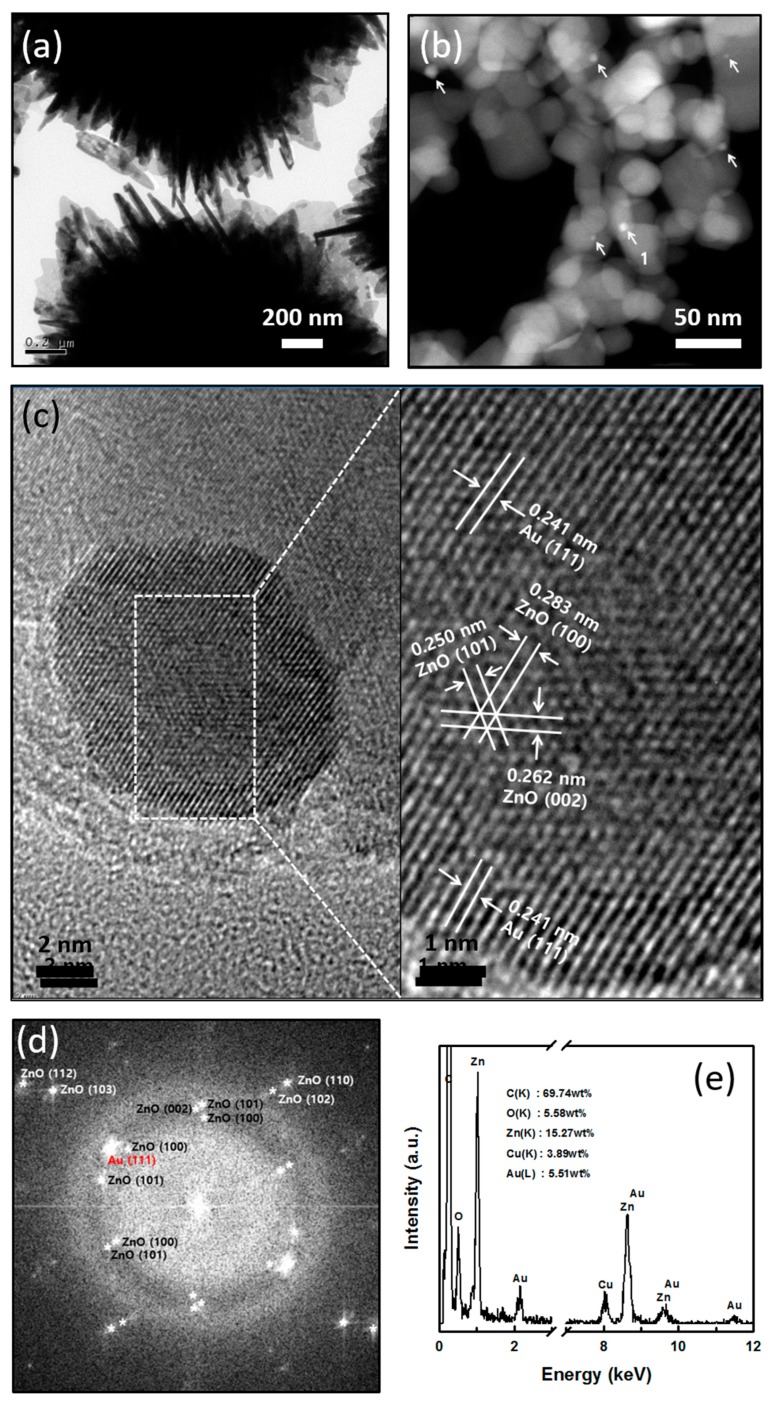
(**a**) TEM image of pristine cotton ball-like Au/ZnO; (**b**) HAADF-STEM image of crushed cotton ball-like Au/ZnO; (**c**) HRTEM image (The inset shows the HRTEM image of the marked area by the square 1) of a single particle as indicated by arrow 1 in Figure 4b; (**d**) Fourier transform image; and (**e**) the EDS spectrum for the dotted square in Figure 4c, respectively.

HRTEM, in conjunction with HAADF-STEM, which is a powerful technique for determining the structural composition of NPs and allows for direct correlation of the optical response with their structural composition, was used to see the Au particles in the cotton ball-like Au/ZnO. The pristine cotton ball-like Au/ZnO sample was dispersed in ethanol, followed by droplet deposition directly on a TEM grid. As shown in Figure 4a, the particles in the pristine sample were too thick to exceed the penetration depth of electrons in the TEM, making detailed imaging difficult. To obtain a specimen (typically less than 100 nm thick for good imaging), the pristine Au/ZnO particles were crushed via ultrasonication and then used for further TEM characterization. Clearly, nanosized particles can be seen as bright objects from the STEM image of the crushed sample (indicated by arrows in Figure 4b). Particles, with an average size of 4.6 nm, were obtained, which is in good agreement with the crystallite size obtained from the XRD (Figure 2b). From the HRTEM image (Figure 4c), the measured interplanar spacing for the lattice fringes was 0.241 nm, which is associated with the (111) plane of the cubic face-centered fcc gold crystals. This indicates clearly the presence of Au particles in the Au/ZnO synthesized in a micro-reactor. We also found that the lattice planes are not continuously extended to the gold at the central region of the particle, which could be due to the dislocations or stacking faults in the Au crystal structure. The corresponding fast Fourier transform analysis (for the dotted square in Figure 4c) indicates the coexistence of Au and ZnO in the central region, with a single crystalline nature. The nanosized particles were also determined to be monometallic from the EDS analysis (Figure 4e). The X-ray signals of the Au-L1 line were clearly observed at ~9.7 and 11.4 keV. Taken together, these results indicate that the Au NPs in the Au/ZnO are intercalated during synthesis in the micro-reaction system and that the Au NPs may interrupt the formation of larger ZnO structures. The formation pathway of the core-shell Au/ZnO structure is investigated in detail in Section 3.2.

**Figure 5:**
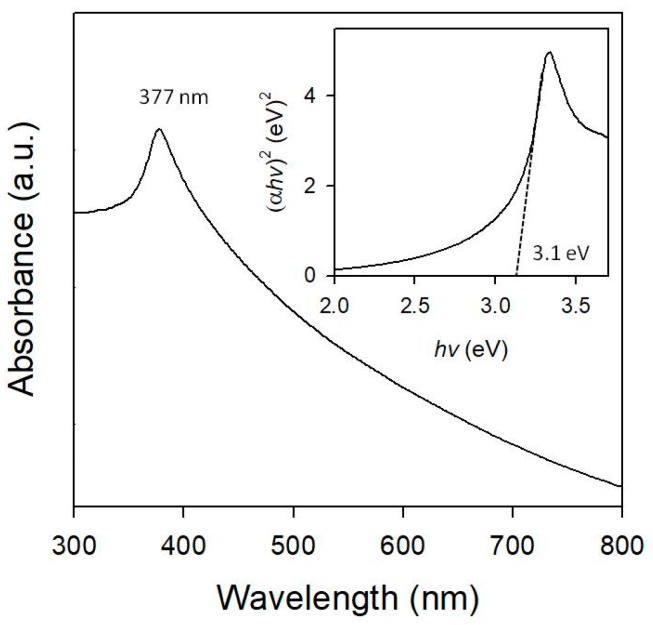
The UV-Vis absorption spectrum at room-temperature of Au/ZnO synthesized in a micro-reactor. Inset of (αhv)^2^ (eV)^2^ vs. photon energy (hv).

UV-Vis spectroscopy was used to study the absorption characteristics of Au/ZnO, and the results are shown in Figure 5. The UV-Vis absorption spectrum of the cotton ball-like Au/ZnO structure was analogous to that of pure ZnO [33,50]. We observed the color of the Au-ZnO-CP sample changed to purple, which is characteristic of the surface plasmon resonance (SPR) of metallic Au NPs. In contrast, the cotton ball-like core-shell Au/ZnO showed a white color (such as ZnO), with a slight yellow hue. The UV-Vis absorption spectrum indicates that no absorption arising from the Au NPs-related SPR peak [51,52] was observed in the visible region. This is probably due to the relatively low concentration of very small Au NPs embedded in the ZnO becoming too broad in the absorption spectrum or being lost in the background that they partly overlapped with the ZnO absorption bands. Broad absorption of Au/ZnO in the visible region demonstrates that Au NPs in ZnO exhibit good optical property and, therefore, can be used as an efficient photocatalyst under visible light irradiation. The Au/ZnO exhibits a peak absorption peak at 377 nm. Compared to bulk ZnO (367 nm, 3.3 eV), the cotton ball-like Au/ZnO structure red-shifted by 10 nm, which is associated to the existence of Au NPs and/or their unique microcrystalline morphology [53]. The estimated band gap energy of Au/ZnO is around ~3.1 eV (inset of Figure 5).

### 3.2. Formation Pathway of Core-Shell Au/ZnO

**Figure 6:**
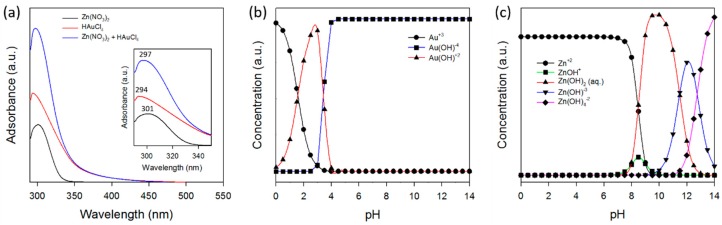
(**a**) UV-Vis spectra of Zn(NO_3_)_2_ and HAuCl_4_ solutions as precursors. Speciation diagram of (**b**) HAuCl_4_ and (**c**) Zn(NO_3_)_2_ precursors in solution with pH.

Mechanistic understanding is needed to produce Au/ZnO of sufficient quality for applications. Figure 6a shows a comparison between the UV-Vis absorption spectra of the Zn(NO_3_)_2_ and HAuCl_4_ in the precursor solutions, and their mixed solutions. The UV-Vis absorption spectra characterizing the solution containing the Zn(NO_3_)_2_ and HAuCl_4_ species consisted of strong bands at 301 nm and 294 nm, which can be assigned to the NO_3_^−^ [54] and AuCl_4_^−^ [55] ions, respectively. The formation of a new chemical phase was not observed in the solutions during the mixing of the selected precursors.

The possible mechanism of core-shell Au/ZnO formation in the micro-reactor under hydrothermal conditions was investigated by using the Visual MINTEQ equilibrium speciation program, where pH adjustment is critical. Figure 6b,c shows the pH-dependent speciation diagram of Au and Zn complexes in the solution, respectively. Again, at room temperature, Zn(NO_3_)_2_ and HAuCl_4_ ions exist in Zn^2+^ + NO_3_^−^ and AuCl_4_^−^ + 2H^+^ forms in the precursor solution, which are transformed to [Zn(OH_2_)_4−n_(OH)_n_]^2−n^ and [Au(OH)_n+1_Cl_3−n_]^−^, respectively, with an increase in pH. The pH of the solution is 9.5 when 0.1 M of Na_2_CO_3_ and 0.05 M of Zn(NO_3_)_2_ and 4.2 × 10^−4^ M of HAuCl_4_ are mixed at 25 °C.

In principle, the crystal growth process includes nuclei and growth. The micro-reaction system allows for precise control of reaction conditions and even allows for the separation of nucleation and growth stages, which enables the formation pathway for these Au/ZnO structures. The separation of nucleation and growth, which cannot be achieved in conventional batch reactors, would control the formation pathway of the crystal growth due to the precise controllability of reaction conditions. At the early stage in a micro-reactor (mixing zone), we observed that a turbid white color was immediately/consistently formed in the tubing at room temperature. This is an insoluble Zn(OH)_2_ species and it has been used as a seed to grow ZnO as we discussed in the prior work [41]. This also indicates that a larger quantity of Zn(OH)_2_ and a smaller quantity of the growth unit, Zn(OH)_4_^−2^, are formed. At the same time, AuCl_4_^−^ ions are fully hydrolyzed to Au(OH)_4_^−^ ions. The isoelectric point of Zn(OH)_2_ is at a pH of 7.8, which indicates that the positively charged species (i.e., Zn(OH)_2_^+^) dominates because of the protonation of the surface hydroxyl group. Thus, electrostatic adsorption of the anionic Au(OH)_4_^−^ ions occurs at the surface of the Zn(OH)_2_ seed as a direct anion exchange, and is continuously delivered to the growth zone. The reaction at an elevated temperature can take place sequentially:

Zn(OH)_2_ + 2OH^−^ ↔ Zn(OH)_4_^−2^

**Figure 7:**
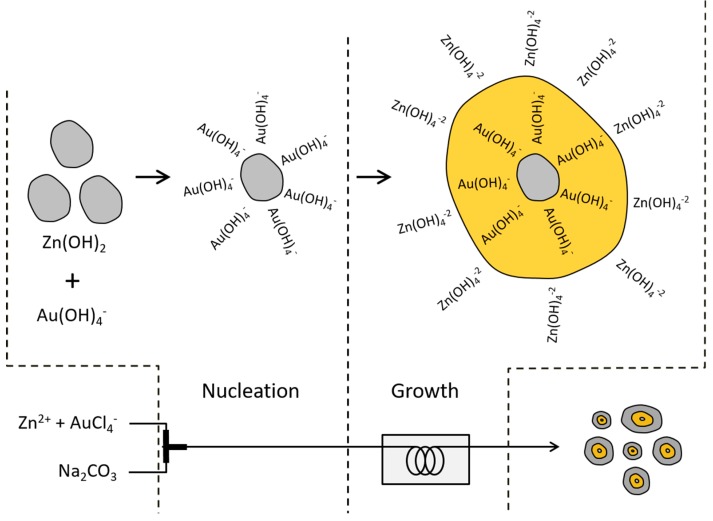
Schematic growth diagram of the core-shell Au/ZnO structure synthesized in a micro-reactor in this study.

This implies that a smaller quantity of Zn(OH)_2_ and a larger quantity of the growth unit, Zn(OH)_4_^−2^, are formed in the solution at the growth zone. Thus, in the hydrothermal process, there is enough growth unit to make ZnO grow from the circumference of the complex, Zn(OH)_2_-Au(OH)_4_^−^, resulting in the core-shell Au/ZnO structure after calcination (Figure 7). The more obvious white color in the growth zone as compared to the nucleation zone indicates the growth of ZnO.

On the other hand, when using the CP synthesis method, the precursor solution is a mixture of zinc hydroxides with Au(OH)_3_, which was formed by the neutralization of metal salts with alkali and deposited on the support surface at a growth temperature. After calcination, hydroxides of the supports are converted to metal oxides (ZnO), and Au(OH)_3_ is reduced to metallic Au, where Au NPs supported on the ZnO are obtained [42]. Therefore, the advantage of the micro-reactor in the synthesis of Au/ZnO is in the ability to mix the precursor solution with the alkali solution instantly, leading to Zn(OH)_2_ as ZnO nuclei uniformly in the solution.

### 3.3. Photocatalytic Activity of Methylene Blue Degradation

**Figure 8:**
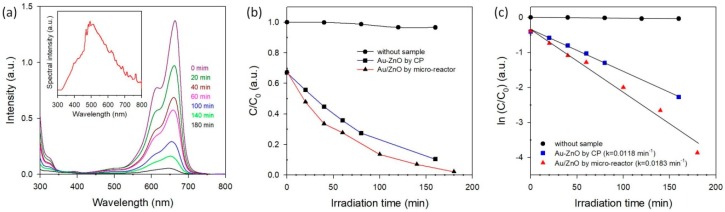
Photocatalytic activities under simulated sunlight of the MB solution without sample, with Au-ZnO by CP, and with Au/ZnO by micro-reactor; (**a**) absorption spectra of the MB solution (10 mg·L^−1^) with irradiation time in the presence of Au/ZnO (The insert shows the used solar spectrum); (**b**) photocatalytic degradation of MB with irradiation time; and (**c**) a plot of ln (C/C_0_) versus irradiation time. First-order degradation rate constants were obtained by plotting the natural logarithm of the absorbance versus irradiation time.

Photocatalytic activities of the cotton ball-like Au/ZnO were investigated by the degradation of MB solution under simulated sunlight. The evolution of the absorption spectra during the photocatalytic degradation of MB with Au/ZnO is shown in Figure 8a. It is well-known that pure ZnO with a wide band gap (3.3 eV for anatase and 3.0 eV for rutile) can be excited mostly by UV light. Hence, little to no photon-induced electron-hole pairs can be generated on the ZnO band-gap under visible light irradiation, leading to the fact that the photo-degradation of MB over ZnO is negligible. In contrast, it can be clearly seen that fast degradation of MB is achieved by adding a cotton ball-like Au/ZnO, indicating an existence of Au NPs in ZnO. Interestingly, a cotton ball-like core-shell Au/ZnO shows a higher photocatalytic activity than the Au-ZnO synthesized by the co-precipitation method (where most Au NPs exist on the surface of ZnO) [42], as shown in Figure 8b. This indicates that the interaction between the Au NPs and ZnO at the interfaces plays an important role in the photocatalytic activity. The kinetic linear fitting results (Figure 8c) reveal that the kinetic data of the photocatalytic degradation of MB match the first-order reaction kinetic model well. The reaction rate constant (k) values corresponding to various photocatalysts are summarized visually in Figure 8c. Specifically, the reaction rate constant catalyzed by the Au/ZnO synthesized in a micro-reactor is 1.6-fold of that of the reaction catalyzed by the Au-ZnO synthesized by the co-precipitation method.

It is widely regarded that Au NPs in metal oxides absorb strongly in the visible light region due to the SPR effect, and this SPR effect has a strong influence on visible-light driven photocatalysis with noble metal NPs on semiconducting materials [56,57,58]. However, no obvious SPR is observed with the cotton ball-like core-shell Au/ZnO photocatalyst synthesized in a micro-reactor, indicating that SPR does not affect the photocatalytic activities for the core-shell Au/ZnO in this work; Au NPs, which could efficiently act as a sink for photo-induced charge carrier separation, also promote interfacial charge-transfer processes [2,3].

## 4. Conclusions

Here, we have introduced a simple and scalable method to synthesize noble metal/metal oxide particle systems for efficient photocatalysts. From various measurements, it is demonstrated that a cotton ball-like Au/ZnO with a core-shell structure was synthesized in a continuous-flow micro-reactor. The existence of Au NPs in the cotton ball-like core-shell Au/ZnO structure was confirmed by HRTEM, XRD, and ICP-AES analysis. The formation pathway of the core-shell Au/ZnO structures was discussed with the pH-dependent speciation diagram. Compared to conventional Au-ZnO systems, higher photocatalytic performance was observed for the cotton ball-like core-shell Au/ZnO structure, where no SPR effect on the photocatalytic activity of dye degradation was observed. Taken together, such micro-reactor-based processes could provide a scalable platform for synthesizing higher-order nanostructures, such as metal/metal oxide core/shell or metal and alloy NPs systems under ambient conditions.
